# Vitamin E and Non-alcoholic Fatty Liver Disease: Investigating the Evidence Through a Systematic Review

**DOI:** 10.7759/cureus.72596

**Published:** 2024-10-29

**Authors:** Mahlet Abera, Suchith B Suresh, Aparna Malireddi, Sruthi Boddeti, Khutaija Noor, Mehwish Ansar, Iana Malasevskaia

**Affiliations:** 1 Medicine, Saint Paul's Millennium Medical College, Addis Ababa, ETH; 2 Medicine, California Institute of Behavioral Neurosciences & Psychology, Fairfield, USA; 3 Internal Medicine, California Institute of Behavioral Neurosciences & Psychology, Fairfield, USA; 4 Internal Medicine, Andhra Medical College, Visakhapatnam, IND; 5 Psychiatry, Harvard Medical School, Boston, USA; 6 Neuropsychiatry, PsychCare Consultants Research, Saint Louis, USA; 7 Internal Medicine, Shadan Institute of Medical Sciences, Hyderabad, IND; 8 General Surgery, Wirral University Teaching Hospital, Wirral, GBR; 9 General Surgery, California Institute of Behavioral Neurosciences & Psychology, Fairfield, USA; 10 Obstetrics and Gynecology, Private Clinic 'Yana Alexandr', Sana'a, YEM; 11 Research and Development, California Institute of Behavioral Neurosciences & Psychology, Fairfield, USA

**Keywords:** adults, liver enzymes, liver histology, non-alcoholic fatty liver disease, vitamin e

## Abstract

Nonalcoholic fatty liver disease (NAFLD), now recognized as metabolic dysfunction-associated steatotic liver disease (MASLD), is a common chronic liver condition characterized by hepatic steatosis and inflammation, with an increased risk of developing fibrosis and cirrhosis. This systematic review aims to evaluate the effects of vitamin E supplementation on liver enzymes and histological features in patients with NAFLD/MASLD. We conducted a comprehensive literature search from July 14, 2024, to July 30, 2024, across multiple databases, including PubMed, MEDLINE, ScienceDirect, Europe PMC, the Cochrane Central Register of Controlled Trials (CENTRAL), ClinicalTrials.gov, and Embase. The included studies consisted of randomized controlled trials (RCTs), clinical trials, and observational studies that evaluated the effects of vitamin E on liver enzymes (ALT and AST) and histological outcomes (steatosis, inflammation, and fibrosis) in adults diagnosed with NAFLD or NASH. A total of 11 studies were analyzed in the final review. Our results indicate that vitamin E supplementation significantly reduces serum aminotransferases and improves histological parameters such as steatosis and inflammation. However, the evidence regarding its efficacy in enhancing fibrosis remains inconclusive, highlighting a significant gap in the current literature. Vitamin E substantially improves liver function and reduces inflammation in patients with NAFLD/MASLD. However, its role in resolving fibrosis remains uncertain, indicating the need for more rigorous, long-term studies to understand its potential to reverse or halt fibrosis progression fully. Further research is required to determine its long-term effects and therapeutic potential, particularly in advanced stages of liver disease.

## Introduction and background

Nonalcoholic fatty liver disease (NAFLD), now renamed as metabolic dysfunction-associated steatotic liver disease (MASLD), is one of the alarmingly increasing chronic liver diseases worldwide, impacting approximately 32% of adults [1]. NAFLD exhibits liver histological features similar to alcohol-induced liver injury. However, it occurs in individuals who consume minimal or no alcohol (less than 20 g/day for women and less than 30 g/day for men). NAFLD is commonly linked to insulin resistance and is frequently accompanied by at least one characteristic of metabolic syndrome, such as obesity, elevated blood sugar levels, high triglycerides, low HDL cholesterol, or high blood pressure [2]. It includes a range of liver conditions, from simple fat accumulation (hepatic steatosis) to more severe forms like inflammation (nonalcoholic steatohepatitis), which can further advance to fibrosis, cirrhosis, or liver cancer [1,3,4]. NAFLD prevalence worldwide has sharply increased from 26% before 2005 to 38% in studies since 2016. The more severe form, NASH, is quickly becoming a leading cause of liver transplants in the United States and other regions. Predictions indicate that cirrhosis, liver failure, liver cancer, and deaths linked to NAFLD will continue to rise through 2030 [5].

The development of NAFLD is complex, with insulin resistance being a key factor. According to the "two-hit" hypothesis, the first "hit" is insulin resistance, which causes fat buildup in the liver (steatosis). In contrast, the second "hit" involves oxidative stress, inflammation, or mitochondrial dysfunction, leading to liver damage and promoting the progression from NAFLD to NASH [2]. Management of NAFLD primarily involves lifestyle interventions, such as a healthy diet and regular exercise to improve both liver and cardiovascular health. In addition, medications for comorbidities like type 2 diabetes mellitus (T2DM) and obesity can also benefit liver health [3].

Vitamin E, a potent antioxidant, has emerged as a therapeutic option, particularly for non-diabetic patients with NAFLD. Several studies have shown that vitamin E supplementation can significantly improve liver enzyme levels, aspartate aminotransferase (AST), and alanine aminotransferase (ALT), reduce hepatic steatosis, and improve liver inflammation. However, evidence regarding its effect on fibrosis is inconsistent, likely due to differences in study designs, patient populations, treatment durations, and diagnostic methods. Fibrosis, being a more advanced liver condition, may require longer treatment periods for improvement, and this variability has led to a gap in understanding its long-term effects. More rigorous, standardized studies are needed to clarify the role of vitamin E in fibrosis treatment [6,7].

This systematic review aims to analyze the available data on the role of vitamin E in improving liver enzymes, hepatic steatosis, and histological features of NAFLD. By addressing this gap, the review seeks to clarify vitamin E's therapeutic potential and its place in managing this increasingly prevalent disease.

## Review

Methods and materials

This systematic review investigates the role of vitamin E supplementation in NAFLD. The primary objectives were to assess the effects of vitamin E on liver enzyme levels (ALT and AST) and liver histology (steatosis, inflammation, and fibrosis). To do this systematic review, we adhered to the Preferred Reporting Items for Systematic Review and Meta-Analyses (PRISMA) 2020 guidelines [8].

Eligibility Criteria and Study Selection

We aimed to identify studies assessing the effects of vitamin E supplementation in NAFLD patients. The inclusion criteria were: (1) adult patients aged over 18 years diagnosed with NAFLD or NASH, irrespective of gender or ethnicity; (2) the intervention group receiving vitamin E (tocopherol, tocotrienol, or combined forms), in varying doses, duration, or routes of administration, with either placebo or another intervention as the comparison group; (3) studies evaluating the effect of vitamin E on at least one treatment outcome. The primary outcomes assessed were liver enzymes (ALT and AST) and histological alterations based on the NAFLD activity score (NAS), which includes steatosis, lobular inflammation, hepatocellular ballooning, and fibrosis score; and (4) study designs such as randomized controlled trials, clinical trials, cohort studies, and observational studies in English, regardless of the publication year or number of participants.

Exclusion criteria were also established to maintain the integrity of the review: (1) studies involving children or adolescents were excluded; (2) patients with other liver-related conditions, such as alcoholic steatohepatitis, viral or autoimmune hepatitis, drug-induced liver disease, cholestatic disorders, Wilson's disease, or chronic hepatitis due to hormonal, genetic, or hereditary causes were also excluded; (3) animal studies, editorials, reviews, opinion pieces, and abstracts without full-text articles were not considered. There were no restrictions on the publication period.

Literature Search Strategy

We conducted an extensive literature search using electronic databases from July 14, 2024, to July 30, 2024. The databases searched included PubMed, ScienceDirect, Europe PMC, the Cochrane Central Register of Controlled Trials (CENTRAL), ClinicalTrials.gov, and Embase. The objective was to identify studies that evaluated the effect of vitamin E supplementation on liver enzymes and histology in NAFLD.

Our search included a combination of keywords and MeSH (Medical Subject Headings) terms, including "nonalcoholic fatty liver disease," "liver steatosis," "fatty liver," "vitamin E," "alpha-tocopherol," and "tocotrienol" (Table 1).

**Table 1:** Search strategies CENTRAL: Cochrane Central Register of Controlled Trials; Europe PMC: Europe PubMed Central; Embase: Excerpta Medica Database; MeSH: Medical Subject Headings; NAFLD: Nonalcoholic fatty liver disease; NASH: Nonalcoholic steatohepatitis.

Search strategy	Databases/ registers	Number of articles before/after filters	Filters used
"vitamin e*"[All Fields] OR "alpha tocopherol"[All Fields] OR "alpha tocopherol"[All Fields] OR "gamma-Tocopherol"[All Fields] OR "gamma-Tocopherol"[All Fields] OR "Tocopherol"[All Fields] OR "Vitamin E"[MeSH Terms]) AND ("non alcoholic fatty liver disease*"[All Fields] OR "NAFLD"[All Fields] OR "Non-Alcoholic Steatohepatitis"[All Fields] OR "NASH"[All Fields] OR "Metabolic-associated fatty liver disease"[All Fields] OR "MAFLD"[All Fields] OR "Non-alcoholic Fatty Liver Disease"[MeSH Terms]) AND ("liver enzymes*"[All Fields] OR "Alanine Aminotransferase"[All Fields] OR "ALT"[All Fields] OR "Aspartate Aminotransferase"[All Fields] OR "AST"[All Fields] OR "Liver Function Tests"[MeSH Terms]) AND ("Liver Histology"[All Fields] OR "Hepatic Steatosis"[All Fields] OR "NASH score"[All Fields] OR "Liver biopsy"[All Fields] OR "Liver fibrosis"[All Fields] OR "Fatty Liver"[MeSH Terms])“vitamin E" AND "Non-Alcoholic Fatty Liver Disease” OR "Non-Alcoholic Steatohepatitis” AND "Liver Function TestS" AND "hepatic steatosis"	PubMed	127/35	Age (19+ years), humans, full text, and English studies
("Vitamin E" AND "Nonalcoholic fatty liver disease") AND (TITLE:"Vitamin E AND Nonalcoholic fatty liver disease") AND (IN_EPMC:y) AND (OPEN_ACCESS:y) AND (HAS_FT:Y)	Europe PMC	2/2	Full-text research articles
“vitamin E" AND "Non-Alcoholic Fatty Liver Disease” OR "Non-Alcoholic Steatohepatitis” AND "Liver Function Tests" AND "hepatic steatosis"	ScienceDirect	2054/152	Research articles in medicine and dentistry, English, open access, and open archive
1. "Vitamin E" OR Tocopherol NEAR "α-Tocopherol" OR "γ-Tocopherol" 2. "Nonalcoholic Fatty Liver Disease" OR "NAFLD" NEAR "Non-Alcoholic Steatohepatitis" OR "Fatty Liver Disease" OR "Hepatic Steatosis" 3. Adults OR "Adult Patients" OR "Middle-Aged" 4. "Liver EnzymeS” OR "Alanine Aminotransferase" OR “ALT” OR "Aspartate Aminotransferase” OR “AST” OR "Liver Function Tests" 5. “Liver Histology” OR “Hepatic Steatosis” OR “NASH score” OR “Liver biopsy” OR "Fatty Liver" 6- #1 AND #2 AND #3 7. #6 AND #4 AND #5	Cochrane Library (CENTRAL)	83/74	Only clinical trials excluding reviews, protocols, editorials, and clinical answers
"Non-alcoholic Fatty Liver Disease" | "Vitamin E" OR Tocopherol | Completed studies | Adult (18 - 64), Older adult (65+) | Interventional, Observational studies | Studies with results	Clinical trials.gov Register	38/7	Age (>18 years), interventional, observational, and completed with results
('vitamin E'/exp OR 'tocopherol'/exp OR "Vitamin E" OR Tocopherol OR "Alpha-tocopherol" OR "α-Tocopherol" OR "γ-Tocopherol") AND ('nonalcoholic fatty liver disease'/exp OR 'nonalcoholic steatohepatitis'/exp OR "Non-Alcoholic Fatty Liver Disease" OR NAFLD OR "Nonalcoholic Fatty Liver Disease" OR "Non-Alcoholic Steatohepatitis" OR NASH OR "Fatty Liver Disease" OR "Nonalcoholic Steatohepatitis" OR "Hepatic Steatosis") AND ('liver function test'/exp OR 'alanine aminotransferase'/exp OR 'aspartate aminotransferase'/exp OR "Liver Enzymes" OR "Liver Function Tests" OR LFTs OR "Alanine Aminotransferase" OR ALT OR "Aspartate Aminotransferase" OR AST OR "Serum Transaminases")AND ('liver biopsy'/exp OR 'liver histology'/exp OR 'liver fibrosis'/exp OR "Liver Histology" OR "Liver Biopsy" OR "Hepatic Histology" OR Steatosis OR "Hepatic Steatosis" OR Inflammation OR "Hepatic Inflammation" OR Fibrosis OR "Liver Fibrosis" OR "Hepatic Fibrosis") AND('adult'/exp OR 'middle aged'/exp OR 'elderly'/exp OR Adults OR "Adult Patients" OR "Middle-Aged" OR Elderly OR Seniors) AND #2 AND ('fatty liver'/dm OR 'nonalcoholic fatty liver'/dm) AND ('clinical article'/de OR 'comparative study'/de OR 'controlled study'/de OR 'cross sectional study'/de OR 'human'/de OR 'observational study'/de OR 'randomized controlled trial'/de OR 'systematic review'/de) AND ([adult]/lim OR [middle aged]/lim OR [young adult]/lim) AND 'article'/it	Embase	339/84	Age > 18 years, RCT, clinical trial, observational, and comparative study

Data Collection and Analysis

All data collected from the six electronic databases and key characteristics of the identified studies were included: study attributes (authors, publication year, study design), population, and treatment specifics (type, dosage, and duration). The primary outcomes assessed were liver enzyme levels and liver histological features. The definitions of the outcome measurements are detailed in Table 2.

**Table 2:** Definition of outcomes ALT: Alanine aminotransferase; NAFLD: Nonalcoholic fatty liver disease; NASH: Nonalcoholic steatohepatitis.

Outcome	Definition and Measurement/Grading
ALT and AST	Enzymes found in the liver and other tissues and elevated ALT levels are key markers of liver injury in NAFLD.
Hepatic steatosis	Accumulation of fat in liver cells, a hallmark of NAFLD graded as part of the NAFLD activity score (NAS): 0 (none) to 3 (severe).
Lobular inflammation	Presence of inflammatory cells in liver lobules, indicating ongoing liver damage graded in NAS: 0 (none) to 3 (severe).
Hepatocyte ballooning	Swelling of liver cells due to damage, characteristic of more severe NAFLD (NASH) graded in NAS: 0 (none) to 2 (severe).
Fibrosis	Buildup of scar tissue in the liver due to chronic inflammation; can progress to cirrhosis graded using NAS or fibrosis staging systems: F0 (none) to F4 (cirrhosis).

Screening and Quality Assessment

Screening and quality assessment were performed using the Rayyan application [9]. Duplicates were removed, and collected records were screened in two steps: (1) title and abstract screening and (2) full-text screening using inclusion and exclusion criteria. All data were reviewed independently by MA and SB. We resolved any conflicts through discussion and consensus.

The included RCT studies were evaluated for quality using the Cochrane Risk of Bias (ROB 2) tool, which assesses five domains: random sequence generation, deviations from intended interventions, missing outcome data, measurement of the outcome, and selection of the reported result [10]. Additionally, the Newcastle-Ottawa Scale was applied to the included cohort study to evaluate the quality based on three main criteria: selection of study groups, comparability of groups, and assessment of outcomes [11].

Results

**Figure 1:**
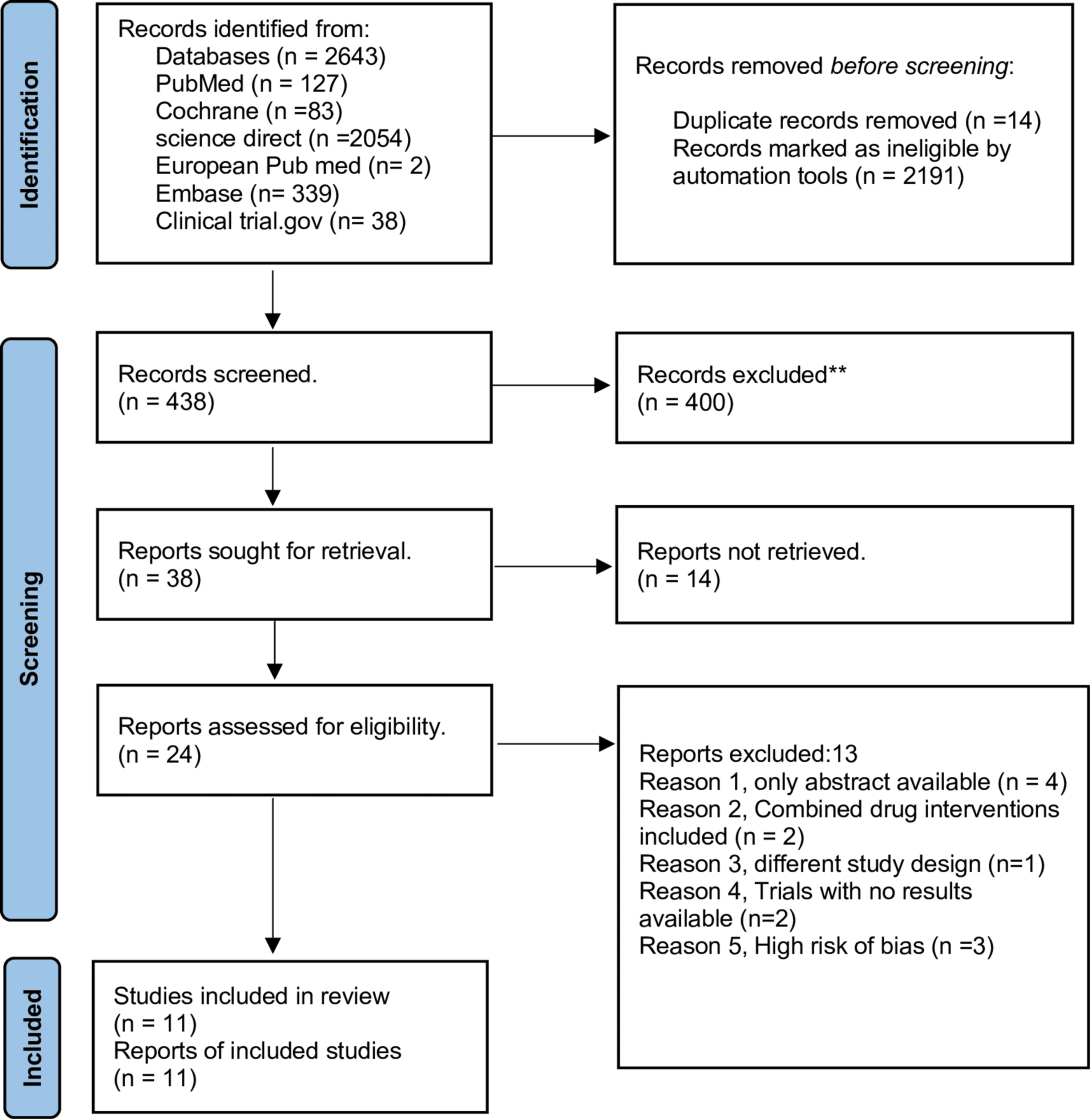
PRISMA flow diagram PRISMA: Preferred Reporting Items for Systematic Reviews and Meta-Analyses.

A total of 2,643 records were identified after a thorough search strategy from multiple databases. After removing duplicates and conducting in-depth screening, 24 studies were eligible. Finally, 11 studies met the inclusion criteria and were included in our final review. The steps followed for selecting included records are represented in the PRISMA flow diagram (Figure 1).

Among the included RCTs, five of them were rated as low risk across all five domains, reflecting well-conducted studies with minimal concerns. Four of the studies were flagged with some concerns, displaying concerns in the randomization process, and missing or unclear outcomes. In summary, while most studies demonstrate strong methodological quality, a few present moderate concerns, particularly regarding randomization processes and outcome reporting, which should be considered when interpreting their results.

Three RCTs were excluded from the systematic review due to a high risk of bias, which compromised the reliability of their findings. The study by Kedarisetty et al. was excluded because of its open-label design and issues with handling missing data, which could have affected the validity of liver histology and biochemical outcomes [12]. Similarly, Parikh et al. were excluded due to the high risk of bias related to the open-label design, lack of allocation concealment, and inadequate blinding of outcome assessors, potentially influencing subjective outcomes [13]. Lastly, the study by Yakaryilmaz et al. was excluded for lacking randomization, allocation concealment, and blinding, which will affect the reliability of its results [14]. The details of the quality assessment are provided in Table 3.

**Table 3:** Risk of bias assessment of randomized clinical trials: Cochrane Risk of Bias tool (RoB 2) Note: RoB 2 domains: (D1) randomization process, (D2) deviations from intended interventions, (D3) missing outcome data, (D4) measurement of outcome, and (D5) selection of reported results. 🟢 indicates low risk of bias; 🟡 indicates some concerns; and 🔴 indicates high risk of bias.

Study/Year	D1	D2	D3	D4	D5	Overall Risk
Kedarisetty et al., 2021 [12]	🟢	🔴	🟡	🟢	🟢	🔴
Parikh et al., 2016 [13]	🟢	🔴	🟢	🟢	🟢	🔴
Yakaryilmaz et al., 2007 [14]	🔴	🟡	🟢	🔴	🟡	🔴
Sanyal et al., 2010 [15]	🟢	🟢	🟡	🟢	🟢	🟡
Magosso et al., 2013 [16]	🟢	🟢	🟢	🟢	🟢	🟢
Polyzos et al., 2017 [17]	🟢	🟡	🟢	🟡	🟢	🟡
Pervez et al., 2018 [18]	🟢	🟢	🟢	🟢	🟢	🟢
Bril F et al., 2019 [19]	🟢	🟢	🟢	🟢	🟢	🟢
Anushiravani et al., 2019 [20]	🟢	🟢	🟢	🟢	🟢	🟢
Rotman et al., 2023 [21]	🟢	🟡	🟡	🟢	🟢	🟡
Fouda et al., 2021 [22]	🟢	🟡	🟢	🟢	🟢	🟡
Pervez et al., 2022 [23]	🟢	🟢	🟢	🟢	🟢	🟢
Chalasani et al., 2023 [24]	🟢	🟢	🟢	🟢	🟢	🟢

Our single included cohort study was assessed using the Newcastle-Ottawa Scale [11], which showed a strong overall score of 8 out of 9.

The Newcastle-Ottawa Scale (NOS) was used to evaluate studies on three broad criteria: selection of study groups, comparability of groups, and assessment of outcomes, with a maximum score of nine stars. Studies with a score of 7 or higher were considered high-quality. Our minimum threshold for inclusion was set at 7 out of 9, ensuring that only studies of good quality were considered. This high score indicates that the study met the quality criteria for selection, comparability, and outcome assessment. It was included in the review due to its good quality and reliability. The assessment indicated that the cohort study adhered to strong methodological standards and offered valuable contributions to the evidence base, thereby supporting the conclusions of the systematic review (Table 4).

**Table 4:** Quality assessment of included cohort study using the Newcastle-Ottawa scale Note: For cohort studies, a maximum of four stars (*) could be awarded for selection, two for comparability, and three for outcome domains. The total score ranges from zero to nine (scores ≥ 7-9, 4-6, and <4 are considered low, intermediate, and high risk, respectively).

Study/Year	Selection	Comparability	Outcome	Overall
Kim et al., 2015 [25]	****	**	**	8/9

Summary of Included Studies

This systematic review evaluated various studies to assess the efficacy of vitamin E in patients with NAFLD, detailing patient demographics, study design, and interventions. The RCTs primarily focused on adult populations, with ages ranging from 18 to 70 years, and included patients diagnosed with NAFLD and NASH. The interventions varied, with vitamin E being a common treatment. Dosages ranged from 200 IU to 1000 mg daily, and treatment durations varied from 12 weeks to 96 weeks. The characteristics of the included studies are summarized in Table 5.

**Table 5:** Summary of included studies RCT: Randomized controlled trial; ALT: Alanine aminotransferase; AST: Aspartate aminotransferase; NAFLD: Nonalcoholic fatty liver disease; NASH: Nonalcoholic steatohepatitis; FLI: Fatty liver index; U/S: Ultrasound; L/S ratio: Liver-to-spleen attenuation ratio; T2DM: Type 2 diabetes mellitus; IU: International unit; UDCA: Ursodeoxycholic acid; SD: Standard deviation; CI: Confidence interval; OR: Odds ratio; FIB-4: Fibrosis-4 score.

Study Author/Year	Type and Duration of the Study	Population	Treatment/Control (Duration)	Liver Enzymes (ALT and AST)	Liver Histology Outcome (Hepatosis, Fibrosis, Ballooning, or Lobular Inflammation)
Sanyal et al., 2010 [15]	A prospective double-dummy, double-blind RCT 2005-2009 USA	Included 247 non-diabetic, adult patients with histologically defined nonalcoholic steatohepatitis	96 weeks, Group 1: vitamin E (n = 84) 800 IU, once daily; Group 2: pioglitazone (n = 80) 30 mg, once daily; Group 3: placebo (n = 83)	Showed early and highly significant decrease in ALT and AST	Showed significantly greater improvement in the histological features of NASH compared to placebo (51% vs. 25% improvement, P < 0.001) Significant reductions were observed in hepatic steatosis (P = 0.005), lobular inflammation (P = 0.02), and hepatocellular ballooning (P = 0.01), but there was no significant improvement in fibrosis scores (P = 0.24).
Magosso et al., 2013 [16]	A randomized placebo-controlled trial, Feb 2008-June 2009, Malaysia	Included 87 untreated hypercholesterolemic adults above the age of 35 with ultrasound-proven NAFLD	12 months, Group 1: vitamin E (n = 30) (mixed tocotrienols) 200 mg, twice per day; Group 2: placebo (n = 34)	No significant improvement	Significant improvement was seen for vitamin E versus placebo for the normalization of hepatic echogenic response (P = 0.039; OR = 2.411; 95% CI)
Polyzos et al., 2017 [17]	A single-center open-label RCT, Jan 2010-Nov 2010, Greece	Age ≥ 18 years; bright liver on U/S, elevated liver test values for at least six months before liver biopsy and biopsy-proven NAFLD	52 weeks, Group 1: vitamin E (n = 17) 400 IU daily; Group 2: vitamin E, 400 IU daily + spironolactone 25 mg daily (n = 14)	No significant improvement	Not assessed
Pervez et al., 2018 [18]	A randomized double-blind control pilot, October 2015-March 2016, Pakistan	Adults > 20 years of age, U/S-proven fatty liver, FLI ≥ 60, and mild to moderate persistent elevation of ALT	12 weeks, Group 1: δ-tocotrienol (n = 31) 300 mg, twice daily; Group 2: placebo (n = 33)	Significant improvement in both AST and ALT	Demonstrated greater efficacy than placebo by reducing the FLI score (P < 0.001). No improvement in hepatic steatosis on ultrasound examination.
Bril et al., 2019 [19]	A randomized double-blind placebo-controlled trial, June 2010-Sep 2016, USA	Adult patients with T2DM and biopsy-proven NASH	18 months, Group 1: vitamin E alone (n = 36), 400 IU twice daily; Group 2: vitamin E + pioglitazone (n = 37); Group 3: placebo (n = 32)	Significant improvement in AST and ALT	Improvements in resolution of NASH compared with placebo: 33% vs. 12%, P = 0.04. No improvement in inflammation, ballooning, or fibrosis.
Anushiravai et al., 2019 [20]	A randomized double-blind placebo, April 2-16 to Oct 2017, controlled trial	Included adult patients of 18 to 65 years old with a probable diagnosis of NAFLD (grades II and III steatosis) on U/S with or without increased levels of AST and ALT	3 months, Group 1: only lifestyle modification 500 kal/day (n = 30); Group 2: vitamin E,400 IU/day + lifestyle modification (n = 30); Group 3: pioglitazone (15 mg/day) + lifestyle modification (n = 30); Group 4: metformin, 500 mg/day lifestyle modification (n = 30); Group 5: silymarin (140 mg/day) + lifestyle modification (n = 30)	Both AST and ALT decreased significantly with (P < 0.001)	Improved steatosis seen
Rotman et al., 2023 [21]	A single-center prospective trial, May 2013-Nov 2016	Adult (age > 18) patients who had two of the following three criteria: (1) imaging consistent with steatosis, (2) elevated ALT, and (3) the presence of metabolic syndrome and/or diabetes.	24 weeks, Group 1: vitamin E (n = 7) 200 IU, once daily; Group 2: vitamin E (n = 6) 400 IU, once daily; Group 3: vitamin E (n = 7) 800 IU, once daily	The absolute change for AST for vitamin E of 200, 400, and 800 IU/day was a decrease of 5.6 (SD 11.1), 1.8 (SD 4.5), and 10.6 U/L (SD 16.7), respectively. The absolute change for ALT for vitamin E of 200, 400, and 800 IU/day was a decrease of 8 (SD 16.7), 8.3 (SD 10.7), and 22 U/L (SD 22), respectively.	The effect of vitamin E on hepatic fat was measured as an absolute change in liver fat percentage. The 200 IU/day group showed a modest reduction of 1.9% (SD 9.6). The 400 IU/day group had a more substantial reduction of 7.6% (SD 3.3). The 800 IU/day group showed a slight reduction of 0.6% (SD 5.2). Higher doses of vitamin E (400 IU/day and 800 IU/day) are more effective in reducing AST and ALT levels, with the 800 IU/day dose showing the greatest overall improvement.
Fouda et al., 2021 [22]	A randomized single-blind study, February 2020-January 2021, Egypt	>18 years old, who had evidence for NASH; persistently elevated ALT > 1.5 times the upper limit of normal, U/S showing fatty infiltration, and histological evidence of NASH after biopsy	3 months, Group 1: vitamin E (n = 34) 400 mg, twice daily; Group 2: UDCA (n = 34) 250 mg, twice daily; Group 3: pentoxyphiline (n = 34) 400 mg, twice daily	The level of AST was reduced by 43%, and the serum level of ALT was reduced significantly by 50%.	Not assessed.
Pervez et al., 2022 [23]	A randomized double-blind active-controlled trial, December 2019-July 2021, Pakistan	Adults aged 20–70 years, with FLI of ≥ 60, L/S ratio of <1.1 (moderate-severe hepatic steatosis), ALT within the reference range, or mildly raised.	48 weeks, Group 1: δ-tocotrienol (n = 50) 300 mg, twice daily, Group 2: α-tocopherol (n = 50) 268 mg, twice daily	Improved AST and ALT significantly	δ-tocotrienol and α-tocopherol exerted equally beneficial effects in terms of improvement in hepatic steatosis and showed significant decreases.
Chalasani et al., 2023 [24]	A multicenter randomized double-blind, placebo-controlled trial, Jan 2020-Sep 2022	205 patients ≥ 18 years of age with a new diagnosis or reconfirmation of previously known fatty liver by imaging (ultrasound or CT or MRI) or by liver biopsy within ≤4 years, Fibroscan CAP score > 300db hepatic fat fraction ≥ 12% by MRI PDFF ALT ≥ 40 U/L	Group 1: Vitamin E (n = 26) 1000 mg once daily; Group 2: placebo (n = 60); Group 3: DHAEE (n = 30) 1.89 g once daily; Group 4: DHAEE, 1.89 g once daily + vitamin E, 1000 mg once daily (n = 61)	Showed a significant decrease in ALT of 16.7 U/L (SD 30.5) and AST of 6.4 U/L (SD 25.1) in patients taking 1000 mg of vitamin E once daily for 6 months	Improved steatosis and inflammations but not fibrosis. For patients who received only vitamin E (1000 mg) for six months, the mean reduction in hepatic fat content was -10.4% (SD 15.7) compared to -8.2% (SD 18.2) in the placebo group. The modest reduction in the FIB-4 score in the vitamin E group suggests a slight improvement in liver fibrosis, decreased by -0.2 units (SD 0.5).
Kim et al., 2015 [25]	A retrospective cohort, May 2003-Dec 2013, South Korea	335 NAFLD patients with metabolic syndrome, >18 years old with elevated baseline (ALT > 40 IU/L) and NAFLD diagnosed by steatosis on ultrasonography	6 months, Group 1: vitamin E (n = 82) 883 IU daily; Group 2: control (n = 250)	Both AST and ALT decreased significantly with P < 0.05 and P < 0.01, respectively	Improved NAFLD fibrosis

Discussion

The present systematic review evaluated the efficacy of vitamin E supplementation in adult patients with NAFLD, with a specific focus on its effects on liver enzymes (AST and ALT), liver histology, and fibrosis. The included studies showed variations in both the dosage and duration of vitamin E supplementation, contributing to differing outcomes. The most used dosage in the trials was 800 IU per day, with durations ranging from 24 to 96 weeks. These higher doses consistently showed significant reductions in liver enzymes (ALT and AST) and improvements in liver histology, specifically in reducing hepatic steatosis, inflammation, and ballooning, though the effect on fibrosis was limited.

Interestingly, lower doses such as 200 IU/day or 400 IU/day, as seen in Polyzos et al. and Rotman et al., also demonstrated improvements in liver enzyme levels but were less effective in influencing liver histology [17,20]. Furthermore, trials using mixed tocotrienols (Magosso et al., Pervez et al., and Pervez et al.) highlighted a range of effects, with improvements in ALT and AST and mild effects on hepatic steatosis, particularly when using doses around 200-300 mg twice daily [16,18,23].

Biochemical Improvement (AST and ALT)

Across multiple studies, vitamin E demonstrated a significant reduction in serum aminotransferases, a key marker of liver injury. In Sanyal et al., vitamin E led to a marked decrease in AST and ALT levels, confirming its beneficial role in reducing liver inflammation [15]. This finding was strengthened in Pervez et al., where both α-tocopherol and δ-tocotrienol showed significant reductions in ALT and AST by up to 32% and 30%, respectively, after 48 weeks of treatment [23]. Similarly, Rotman et al. reported a dose-dependent reduction in liver transaminases, with the 800 IU/day group showing the greatest overall improvement in ALT and AST [21]. These results suggest that vitamin E is effective in improving liver damage in patients with NAFLD, particularly in reducing aminotransferase levels.

Histological Improvement: Steatosis, Inflammation, and Ballooning

Regarding histological improvements, the review reveals consistent reductions in hepatic steatosis and inflammation with vitamin E treatment. In Sanyal et al., vitamin E significantly improved liver histological parameters, including steatosis, lobular inflammation, and hepatocyte ballooning [15]. Similarly, Pervez et al. demonstrated significant improvements in hepatic steatosis and liver-to-spleen attenuation ratios in both tocopherol and tocotrienol groups, further supporting the role of vitamin E in improving liver fat content [23]. Yaron et al. also showed improvements in steatosis, inflammation, and hepatocyte ballooning, indicating that higher doses of vitamin E (400-800 IU/day) are more effective in addressing these histological features [21].

Similarly, Pervez et al. found that while vitamin E significantly reduced aminotransferase levels and the fatty liver index (FLI), there was no significant improvement in hepatic steatosis as assessed by ultrasound [18]. This discrepancy underscores the variability in assessment methods for hepatic steatosis and highlights potential differences in study populations or vitamin E formulations.

Fibrosis: Limited Evidence of Improvement

The effect of vitamin E on fibrosis is less conclusive. While several studies, including Sanyal et al., observed improvements in steatosis and inflammation, they did not show significant changes in fibrosis [15]. Similarly, Bril et al. demonstrated that while vitamin E improved liver steatosis, it did not significantly affect inflammation, ballooning, or fibrosis [19]. This is consistent with the findings of Chalasani et al., where vitamin E showed modest reductions in hepatic fat content but minimal impact on fibrosis scores [24]. The overall evidence for vitamin E’s impact on fibrosis remains limited, suggesting that its primary role may lie in addressing early-stage liver damage rather than reversing advanced fibrosis.

Comparison With Other Evidence

Our study findings on the role of vitamin E in NAFLD management are largely consistent with the other studies reviewed. The systematic review by Usman et al. and review by Perumpail et al. found that vitamin E significantly improved liver enzyme levels, particularly ALT and AST, and reduced inflammation and steatosis [7,26]. These improvements were also reflected in our results, where significant decreases in ALT and AST were observed alongside improvements in steatosis and inflammation. However, similar to our review, the effect of vitamin E on fibrosis was limited, which aligns with the findings of both Usman et al. and Perumpail et al., who highlighted the minimal impact on fibrosis [7,26].

Xu et al. conducted a systematic review supporting these findings, confirming vitamin E's role in biochemical improvement, although it noted limited resolution of fibrosis [27]. Similarly, a review by Mahzar et al. reported improvements in liver enzymes and steatosis but did not demonstrate significant changes in fibrosis [6]. Overall, the consensus across all studies, including our own review, indicates that vitamin E provides substantial benefits in liver function and inflammation in the context of NAFLD/NASH. However, its efficacy in treating fibrosis remains inconclusive and warrants further investigation.

Strengths and Limitations of the Systematic Review and Included Studies

This systematic review demonstrates notable strengths that enhance its credibility and relevance. First and foremost, a comprehensive literature search was conducted across multiple databases. This extensive approach ensured a wide coverage of relevant studies, capturing a broad spectrum of literature related to vitamin E and NAFLD. Moreover, the inclusion of a majority of RCTs, which are considered the gold standard in clinical research, significantly enhanced our review. The systematic review also established clear inclusion and exclusion criteria, focusing on adult populations diagnosed with NAFLD. Furthermore, the focused research questions regarding the effects of vitamin E on liver enzyme levels and histology allowed for targeted conclusions, which can inform clinical practice and guide future research in this area.

Despite its strengths, the systematic review is not without limitations. One significant limitation is the heterogeneity of the included studies. Variability in sample size, intervention dosages, treatment duration, and study populations complicates the synthesis of results. This heterogeneity may limit the generalizability of the findings across different patient demographics and clinical settings.

Another limitation is the inconsistent reporting of histological outcomes. While many studies provided data on liver enzyme levels, fewer reported detailed histological findings, particularly regarding fibrosis. The inclusion criteria also imposed restrictions on language and publication formats, potentially excluding relevant research conducted in other languages or formats, further limiting the breadth of the review.

Clinical Implications

The findings of this systematic review strengthen the potential role of vitamin E as a therapeutic option in the management of NAFLD. Vitamin E supplementation has demonstrated significant benefits in reducing liver enzyme levels (ALT and AST) and improving histological parameters such as steatosis and inflammation. Given the limited pharmacological options currently available for NAFLD, vitamin E represents a promising, well-tolerated treatment that can be integrated into the broader management of the disease, especially in the early stages where inflammation and steatosis are more prominent. Clinicians may consider vitamin E as part of a comprehensive management plan that includes lifestyle modifications. However, the inconclusive evidence regarding its impact on fibrosis necessitates careful patient selection and ongoing monitoring to evaluate long-term outcomes.

The findings of this systematic review strengthen the potential role of vitamin E as a therapeutic option in the management of NAFLD. Vitamin E supplementation has demonstrated significant benefits in reducing liver enzyme levels (ALT and AST) and improving histological parameters such as steatosis and inflammation. Clinicians may consider vitamin E as part of a comprehensive management plan that includes lifestyle modifications. However, the inconclusive evidence regarding its impact on fibrosis necessitates careful patient selection and ongoing monitoring to evaluate long-term outcomes.

Future Research Directions

Future research should prioritize long-term, large-scale RCTs to clarify the effects of vitamin E on liver fibrosis in patients with NAFLD. Investigating optimal dosing regimens, treatment duration, and potential synergistic effects with other therapeutic agents could provide deeper insights into its efficacy. Exploring the potential for vitamin E to be used in combination therapies could significantly enhance its therapeutic effects, particularly in addressing fibrosis, which remains a challenge in NAFLD treatment

In addition to clinical trials, future research should also focus on the mechanisms of action by which vitamin E influences liver histology, particularly fibrosis. Investigating how vitamin E modulates oxidative stress, inflammation, and liver regeneration at a molecular level could reveal pathways for potential therapeutic targets. Furthermore, standardized assessment tools for histological outcomes, especially fibrosis, will be essential for improving comparability across studies and accurately assessing the long-term benefits of vitamin E.

## Conclusions

In conclusion, this systematic review highlights vitamin E's substantial benefits in improving liver enzyme levels and histological features in patients with NAFLD/MASLD. While the evidence supports its role in enhancing liver function and reducing inflammation, the efficacy of vitamin E in treating fibrosis remains uncertain. This knowledge gap indicates the need for further investigation to establish vitamin E's long-term therapeutic potential in advanced liver disease. Addressing these uncertainties will be crucial for optimizing treatment strategies and improving patient outcomes in the growing population affected by NAFLD.
